# Is it the right time for an infant screening for Duchenne muscular dystrophy?

**DOI:** 10.1007/s10072-020-04307-7

**Published:** 2020-02-28

**Authors:** Gian Luca Vita, Giuseppe Vita

**Affiliations:** 1 Nemo Sud Clinical Centre for Neuromuscular Disorders, Messina, Italy; 2 Unit of Neurology and Neuromuscular Diseases, Department of Clinical and Experimental Medicine, University of Messina, Messina, Italy

**Keywords:** Duchenne muscular dystrophy, Early diagnosis, Infant screening, Public health

## Abstract

Newborn screening (NBS) is an essential, preventive public health programme for early identification of disorders whose early treatment can lead to significant reduction in morbidity and mortality. NBS for Duchenne muscular dystrophy (DMD) has been a controversial matter for many years, because of false positives, the lack of effective drugs and the need of more data about screening efficacy. The still high diagnostic delay of DMD and the current availability of drugs such as steroid, ataluren, eteplirsen, golodirsen and forthcoming new drugs, improving the clinical conditions if early started, make appropriate to begin a concrete discussion between stakeholders to identify best practice for DMD screening. A two-step system CK/DNA screening programme is presented to be performed in male infants aged between 6 months and 42 months involving more than 30,000 male infants. Five to eight DMD subjects are believed to be diagnosed. The pilot project would give the opportunity to test in a small population the feasibility of an infant screening programme, which in the near future could be applicable to an entire country.

## Background

Newborn screening (NBS) is internationally recognised as an essential, preventive public health programme for early identification of disorders in newborns that can affect their long-term health. Early detection, diagnosis and treatment can lead to significant reduction in morbidity and mortality and may facilitate informed decision-making for future pregnancies. So far, NBS has been implemented only in very few neuromuscular diseases. Medium-chain acyl-CoA dehydrogenase deficiency, the most common inherited defect of mitochondrial fatty acid oxidation, was included in NBS programmes of many countries since the late 1990s. Measured concentration of acylcarnitines is parameter on which to base a follow-up testing for definitive diagnosis and consequent early treatment including the advice to avoid prolonged fasting and an emergency regimen during intercurrent illness [1]. After introduction of enzyme replacement therapy (ERT) with recombinant human alpha-glucosidase, the first NBS programme for Pompe disease began in Taiwan in 2005 and was then definitely incorporated into the national system in 2008 [2]. Despite some controversies including that (i) most individuals can remain asymptomatic for years or decades and (ii) there is no consensus on when to begin ERT for those with non-infantile forms, some US states began NBS for Pompe disease [3].

The therapeutic scenario of spinal muscular atrophy (SMA) has been recently subverted by introduction of nusinersen [4]. Despite the high cost and different refund policies of health insurances in different countries, its availability is increasing worldwide. Moreover, US Food and Drug Administration (FDA) has just approved SMN1 gene replacement and granted priority review for risdiplam, another SMN2 splicing modifier [4], reinforcing the commitment of health authorities, pharmaceutical companies and advocacy groups to guarantee equity and access to treatment to all patients. The results of clinical trials and real-world evidences support the view that the earlier the treatment is started, the better is the efficacy [5–7]. Nurture trial of nusinersen in pre-symptomatic infants is still ongoing, but positive interim analysis confirms that early diagnosis and treatment have the potential to completely change SMA course. There is at the moment a pressing demand for pilot NBS studies to test the possibility to include SMA NBS in the public health programmes, an ethically and medically obvious target. Pilot NBS experiences of genetic analysis on dried blood spot (DBS) samples have been conducted in Taiwan [8], New York State [9], and Southern Belgium [10], and one is just started in the Italian regions of Tuscany and Lazio.

## Diagnosis and treatment of Duchenne muscular dystrophy

Duchenne muscular dystrophy (DMD) is an X-linked muscle wasting disorder, leading to wheelchair confinement at 8–12 years and development of an associated cardiomyopathy, with an incidence of 1:3600–9300 live male births per year [11]. The onset of muscle weakness is typically in early childhood. Without intervention, few patients survived beyond the second decade of life in the past, but nowadays, improved care, in particular cardiac and ventilatory management, has dramatically increased life expectancy [12–15]. Despite advances in technology and increased availability of genetic testing, the mean age at diagnosis is reported to be around the age of 3.5–5 years with a delay of up to 2 years between the appearance of first symptoms and the diagnosis. The incidental finding of increased creatine kinase (CK) serum levels, a biomarker of membrane fragility and muscle degeneration, may represent the most frequent finding leading to a suspicion of DMD (up to 53% of cases) [16]. In some hospitals or in some countries, CK is measured as a routine blood test or for pre-anaesthesiologic evaluation [17]. Late diagnosis of DMD may cause inappropriate delayed access to optimal Standards of Care (SOC) [18], including steroids, whose benefit is higher if boys are treated earlier [19]. The FDA has also granted full approval for deflazacort in 2017, making it the first glucocorticoid with a labelled indication specifically for DMD. Moreover, a diagnostic odyssey with prolonged and multiple medical evaluations is in itself distressing, is often expensive and prevents parents from accessing genetic counselling and future prenatal diagnosis.

In 2014, the European Medicines Agency (EMA) gave to ataluren a conditional approval for the treatment of ambulatory DMD patients aged 5 years and older with nonsense mutation. Point mutations introducing a premature stop codon into mRNA and therefore causing the translation of a truncated and non-functional dystrophin protein are present in 10–15% of DMD patients [20]. In a few years, ataluren became available as the first approved drug for DMD in more than 25 countries but not in USA. In October 2018, PTC Therapeutics announced preliminary data from the first international drug registry for DMD patients receiving ataluren (Translarna™), highlighting the long-term clinical benefit in delaying irreversible muscle loss when compared with published natural history. In the real-world setting, DMD children and adolescents are continuing to walk years longer than untreated children and are staying more physically able. A time-to-event analysis for loss of ambulation in treated subjects has shown a median age of loss of ambulation of 16.5 years of age (https://www.multivu.com/players/English/8420051-ptc-therapeutics-stride-registry-duchenne-muscular-dystrophy-translarna). Based on the positive results of a phase 2 study in DMD children from 2 to 5 years, in July 2018, EMA has extended the indication of ataluren since the age of 2 years.

Many companies are developing a variety of other drugs for DMD, based on exon-skipping technology and other mechanisms of action [4]. Eteplirsen, commercially available as Exondys 51™ in the USA, obtained in September 2016 a conditional approval by FDA to treat DMD patients amenable to exon 51 skipping (approx. 13% of DMD patients) [21]. In December 2019, FDA approved also golodirsen (Vyondys 53™) to treat DMD patients with a confirmed mutation amenable to exon 53 skipping (approx. 8% of DMD community).

## Lessons from previous experiences of NBS in DMD

The 195th ENMC International workshop on newborn screening for DMD, held in 2012 to discuss pros and cons of an NBS programme to anticipate diagnosis, led to a wait-and-see position, despite the notion that physiotherapy and steroid treatment could be implemented earlier as a result of NBS. The reasons were the lack of effective drugs despite ongoing experimental trials at that time, the belief that NBS programme should be introduced as voluntary screening whose details should be yet evaluated and discussed, and the need to have more data about screening efficacy [22]. At that workshop, the results of a recently published two-tiered CK/DNA NBS approach were presented and discussed [23]. It consisted of i) a DBS CK test in newborn male infants at major birthing hospitals of Columbus and Cincinnati, Ohio, USA after parents’ informed consent; ii) a second CK test by venous blood sample in those resulted with CK ≥ 600 U/l; and iii) DNA screening on DBS in those males with CK ≥ 750 U/l. Lessons learnt were that increased CK level were found in newborns, not confirmed by a second CK test, most likely because of the muscle trauma during progression through the birth canal, and that 308/37,649 had CK levels >750 U/l but only 10 had CK ≥ 2000 U/l. Six males were found to have *DMD* gene mutations, all deletions, 5 out-of-frame and 1 in-frame. All samples with *DMD* mutations had CK values ≥2000 U/l. In the subjects with CK ≥ 2000 U/l without *DMD* mutations, the most common limb-girdle muscular dystrophy (LGMD) genes were investigated and mutations in *DYSF*, *SGCB* and *FKRP* genes were found.

Further interesting results were reported in a study describing 21-year experience of DMD NBS in Wales, UK [24]. The lowest serum CK activity in the boys with confirmed DMD diagnosis was 2442 U/l when measured at the 6- to 8-week follow-up appointment after NBS. Surprisingly, over the 21-year period, 343,025 boys resulted with a CK level < 250 U/l at 5–8 days of life, but 13 were then found to be false-negative. They were diagnosed to have DMD after a level of CK between 6000 and 37,296 U/l was found at the age of 1.4–7.85 years. The authors explained that the false-negative cases simply be due to the insensitivity of the analytical test utilised to screen those samples collected at days 5 to 8 of life.

Recently, the enthusiasm for early DMD diagnosis by NBS increased amongst clinicians and patient associations. Pilot NBS studies have been performed in the Zhejiang Province in China and in Australia, where it is looking for the optimal time, comparing 24–96 h vs 6–7 days vs 6–12 weeks of age [25]. In EU, the general situation of NBS varies a lot, from 5 diseases in France to 9 in UK and 14 in Germany. The most advanced countries are Sweden, Finland, Hungary and Poland, where a range from 22 to 26 diseases are compulsorily screened at birth. Italy profiles itself as a leader amongst European countries with more than 40 diseases, although not all adopted in all regions. No country yet includes DMD.

DMD patients and their parents are strongly in favour of NBS, regardless of whether their diagnosis was made shortly after birth by NBS or after symptom onset. Boys diagnosed by NBS and their parents reported a positive impact of NBS on their quality of life. Expectations are high, and no negative psycho-social impacts of NBS were identified amongst those families who received a diagnosis through NBS [26, 27].

## A proposal of infant screening for DMD

The current availability of drugs such as steroid, ataluren, eteplirsen, golodirsen and forthcoming new drugs, improving the clinical conditions if early started, and the still high diagnostic delay, make appropriate to begin a concrete discussion between DMD experts and advocacy groups to implement pilot screening projects to have additional experience and to identify best practice for DMD screening. A pilot project would give the opportunity to test in a small population the feasibility of a screening programme, which in the near future could be applicable to an entire country. A good screening blood test must be simple to perform, effective, cheap and patient-friendly.

The present two-step system proposal has been selected to receive funding by PTC Innovative Research Funding programme PRIORITY, a 2019 global programme designed with the aim to improve rates of DMD screening in infants and facilitate earlier diagnosis.

### Population

Previous NBS experiences in DMD identified the age older than 2 months as appropriate to avoid false-positive results of increased CK, due to muscle trauma at birth, and to prevent unnecessary family distress. Moreover, DMD screening resulted to be feasible in the paediatric office, during a routine visit, and it has the advantage to give more time to the parents to understand the risks and benefits of DMD screening in a less frantic environment comparing with newborn period [28].

Consequently, DBS will be performed in infants aged at least 6 months. The screening programme will be communicated to the parents during a routine well-child visit by the Italian National Health Service (NHS) family paediatrician. In the provinces of Messina and Catania, Italy, we expect to have approx. 6500 male births/year. According to DMD incidence and since age of symptom onset is mostly between 30 and 42 months, we plan to screen male infants aged between 6 months and 42 months in a 1.5-year period, so a total of more than 30,000 male infants. We expect to find 5–8 DMD subjects. We want to limit the pilot screening study to a relatively small number of participants to be able to test the project and to identify more easily critical issues and areas for improvement. The project has been approved by the local ethics committee. Since we offer to the families the right to have a choice, a signed parental consent is mandatory.

### Information brochure

Parents frequently do not fully understand the implications of being included in an NBS programme. Moreover, in some countries and even in different areas of the same country, several factors such as culture, society, history and religion may influence the opinion of the wider public towards genetic issues. Although diminishing, a discrimination against people with hereditary diseases and disability globally may still exist, so that having a hereditary disease is felt and recognised as a “family’s shame”. In this case, improvement of genetic education and tools for coping with diagnosis may be obtained by adequate information with brochure and families’ participation [17]. For these reasons, a brochure will be prepared together with DMD advocacy groups, describing the disease, its management, how the screening programme will identify infants likely to have DMD or other non-DMD muscle diseases, and its benefits and risks. To allow time for the parents to consider the decision fully and make a reasoned choice, DBS can be collected at the following visit.

### Training, sampling and laboratory methods

Approx. 210 NHS paediatricians, operating in the territory, will participate to the project. They will be visited and trained by nurses involved in the screening programme, to perform blood sampling technique. Small volume of whole blood is collected from a finger, toe or heel prick with the aid of sterile disposable lancet and spotted onto filter paper. DBS cards are then dried at room temperature for 2–3 h in a suitable setting, properly stored and then shipped to analytical laboratory through mail or courier. Before starting the project, experiments will be done to measure long-term stability of CK at room temperature to find the best way of storage and to minimise analyte degradation [29]. The blood spot cards also include demographic information and date and time of collection.

Dried blood spots are punched using a DBS puncher and then processed to measure CK by liquid chromatography mass spectroscopy (LC-MS). LC-MS is a cheap, confident and user-friendly method, widely implemented in paediatric settings and associated to newborn screening programmes, as a part of public health policies [30]. Preliminarily, samples from unaffected, healthy infants will be tested for assay development and validation.

### Follow-up at neuromuscular centre

In one NBS programme, boys with CK levels ≥750 U/l represented 0.8% of the total screened cohort, and in another, approximately 50% of the screen positives (CK ≥ 200 U/l) were transient when repeated at triplicate. However, all boys with confirmed *DMD* mutation had CK levels ≥ 2000 U/l [23, 24, 31]. Therefore, the threshold for DNA testing can be increased, improving the potential cost-benefit ratio, and the following steps are suggested in our proposal:

- Blood samples with CK levels ≥ 1000 U/l are tested for DNA analysis. The multiplex ligation-dependent probe amplification (MLPA) technique will be performed as first approach to detect deletions and duplications in all 79 exons of the dystrophin gene. Since 30% of patients have small mutations which are not detected by MLPA, further analysis will be performed by using the Sanger method/next generation sequencing. In the case of negative results, a next generation sequencing approach will be used by a panel analysis for the screening of other known genes [32, 33]. Communication of DMD or other non-DMD muscle diseases diagnosis will be approached with genetic counselling and providing adequate psychological support. If no gene mutation is confirmed, boys are followed at the neuromuscular centre according to current clinical practice for asymptomatic hyperCKemia [34, 35];
- If CK levels < 1000 but ≥ 250 U/l, parents are asked to allow a retest. If elevated CK is confirmed, above cited guidelines for asymptomatic hyperCKemia will be followed.

Infant screening for hyperCKemia will offer the opportunity to identify not only boys with DMD but also some boys with Becker muscular dystrophy (BMD) and other early-onset treatable myopathies such as Pompe disease [2, 3]. Moreover, identification of DMD/BMD patients will allow to detect also the respective female carriers of their families, allowing a conscious family planning and/or a prenatal diagnosis.

## Discussion

In 2014, ataluren became the first approved drug for DMD patients aged ≥5 years with nonsense mutation, and in 2018, EMA has extended its indication since the age of 2 years. Many companies are developing a variety of other drugs for DMD patients, based on exon-skipping technology and other mechanisms of action. The identification of therapeutic interventions that are more effective as early started and even more in the pre- symptomatic stage has created a pressing demand for NBS studies, in order to detect and start to treat asymfvptomatic individuals and provide the opportunity to slow down disease progression and to reduce morbidity and mortality.

The here present project wants to be a pilot experience about infant screening for DMD to be performed in a territory with approx. 1.7 million inhabitants. It is a feasibility study on a small cohort of subjects to verify different issues. First of all, the problem of false-positive results of increased CK which can be observed in the newborn population is solved by starting DBS at 6 months of age. Consequently, DBS is collected on filter paper by the NHS paediatricians after signed parental consent. Then, the laboratory test is performed at our tertiary neuromuscular centre by using LC-MS. The positive subjects will be invited to a visit at our centre to verify the provisional diagnosis of DMD or other muscle disease and genetic analysis and/or muscle biopsy will be done for confirmation. This will offer the opportunity to diagnose also other muscle disorders with early hyperCKemia. In that event, we have to consider that some of them are treatable such as Pompe disease, but others are untreatable, with early or even later clinical onset. In such cases, genetic counselling, psychological support and follow-up will be offered [36]. On the other hand, we are also conscious that subjects with *DMD* gene mutation-related cardiomyopathy have normal or near normal CK, so that they cannot be identified by this programme. The results of the present screening programme will also allow to evaluate its economic sustainability and the sensibility of CK assay in our population, to better plan future national screening.

The primary goals of early DMD diagnosis are in the best interest of the child and his family, such as to start early intervention and to prevent distressing diagnostic odyssey. Secondary benefits are to provide a prompt family’s genetic counselling, with additional psychological support, to identify DMD carriers and to offer reproductive risk and prenatal diagnosis. Apart from steroid and SOC, early diagnosis would also allow to approach the neurodevelopmental difficulties that are often present in very young DMD boys [37], as well exercise regimes, gradually introduced, to keep muscles supple and prevent or minimise tightness at the joints.

Amongst ongoing DMD clinical trials with innovative medicines, some are based on exon-skipping antisense oligonucleotides (ASO) technology, and at least three are investigating the approach of systemic gene therapy using a micro-dystrophin gene, and in one of them, the age eligible for the study starts at 3 months of age [4]. Dystrophic pathology has been described in DMD foetuses [38], and early pelvic girdle musculature involvement can be identified by magnetic resonance in the first 2 years of life in DMD boys [39]. The therapeutic benefit of early beginning of steroid treatment could be the result of having higher amount of muscle tissue responsive to treatment and lesser fibrosis [19]. This has been supported also with the innovative therapy eteplirsen [40]. Indeed, all these evidences represent the essential prerequisite to implement a specific population screening for early DMD diagnosis.

A very recent evidence-based consensus and systematic review on measures to reduce the time to DMD diagnosis investigated also the attitude of fifteen experts if NBS should be performed. A total of 47% answered “yes”, 47% answered “not sure”, and one answered “no”. The reason for 47% answering “not sure” was mainly related to the absence of available treatments for patients younger than 5 years of age (at the time of investigation ataluren has not been yet approved for children as young as 2 years). Moreover, 75% believed that CK-based NBS would improve standards of care and family planning [41]. NBS has been discussed for many years, but now, with promising treatments already available or on the horizon, it became an even more appropriate matter. We believe that enough reasons support the implementation of NBS programmes for DMD to avoid delays in initiating beneficial treatments.
